# Florence Nightingale and Her “Notes on Nursing.”

**Published:** 1860-04

**Authors:** 


### Florence Nightingale and her "Notes on Nursing."

The *Medical Times and Gazette* makes the following remarks in regard to this eminent lady and her recently published book:

Nursing the sick has been with her a labor of love; the whole tenor of her writings tends to ennoble that vocation, and to redeem it trom the hands of the ignorant, the stupid, and the thoughtless. With noble and most devoted energy she has always endeavored to elevate the calling of the nurse, by bringing thought, intelligence, and study to bear upon her work, and by calling forth the finer feelings of the mind in the exercise of the most humane of all vocations. It is now more than fourteen years since Florence Nightingale began to give her undivided attention to this field of thought and action. Twice has she been in training as a nurse at the Institution of Protestant Deaconesses at Kaiserwerth, on the Rhine. She has studied with the "Sœurs de Charite" in the Hospitals of Paris. She has visited the Hospitals of Berlin, and those of many other towns in Germany. She has visited those of Lyons, Rome, Alexandria, Constantinople, Brussels, and likewise the Hospitals in the chief towns of our own country; but the most extensive sphere of her usefulness, and one where her experience was most matured, was in our Military Hospitals at Scutari and the Crimea, during the Russian war. Thither she was sent by Mr. Sidney Herbert, then and now Secretary at War, who has the honor and merit of having been the first to appreciate, and to put in a position of public usefulness, the singular abilities of Florence Nightingale. What she succeeded in doing at Scutari and elsewhere for our sick and wounded soldiers is now a matter of history. What she has since done in bringing about sanitary improvements in our army has still to be recorded. She has never been at rest since her arrival in this country. With such precedents and with such extensive experience, acquired among scenes of most varied suffering, can any one doubt that a written record of her thoughts and ideas regarding the subject of nursing the sick can be other than of the greatest possible public interest? She has undoubtedly ennobled the calling of the nurse, she has made her vocation a labor of love, and has sacrificed her health in the acquisition of her extensive experience. She has brought to bear upon the subject all the energies of an active and highly cultivated intellect, rendered still more energetic by intense enthusiasm in the work. The asperities of business not unfrequently encountered in the rough walks of life through which she has passed, have been at once smoothed down, or have altogether disappeared through the influence of that remarkable tact with which she is so largely gifted, directed by a mind the most amiable, gentle and refined. We think, therefore, we are justified in the belief we have expressed, that no other living person than Miss Nightingale, could write a book on nursing such as we have now before us. Every line of it, from the preface to the end, rivets the attention, every paragraph is suggestive, every page carries the reader into a world of thought.—*Medical and Surgical Reporter.*

☞ What Sanitary Science has done, and has yet to do, may be gathered from the following facts. The science is quite of modern date; but since the application of its simplest principles, the cleansing of streams, draining of houses, and introduction of pure water, the following evidence of benefits resulting has been given us:—In Liverpool the mortality had fallen from 37 in the 1000 to 27; in Bradford from 28½ to 22; in Gloucester, from 27 to 24. Taking an average of nineteen towns which had been treated in this way, it was found that the death-rate dropped from 28 in the 1000 to 21. Croydon was taken in hand scientifically some time ago; and since then an average of 196 lives have been saved in the town every year! The mortality among the pauper infants and pauper children in the metropolitan unions has been enormously reduced. In the Military School at Chelsea a death-rate of nine in the thousand has been brought down to one of four. The female prisoners at Brixton, who live under sanitary rules, are three times as healthy as the poor needle-women of London; and at Pentonville, notwithstanding the allowance to be made for moral depression, the death-rate is only one-third of that prevailing in populous towns. But still there is a great work to be done; for as we are told, authoritatively, that at least 100,000 persons die annually in these islands at premature periods and by preventible deaths; and at least 1,000,000 more are wasted and debilitated from similar causes. Talk of war, indeed! why what battles or contests ever wrought havoc like this—havoc, be it remembered, not occurring at intervals like an exceptional calamity, but carried steadily and incessantly through the ranks of our population? And we have still to remember the lesson taught us by the Crimean war. In that war we lost altogether 20,800 men; but of this number 5000 only were slain by the enemy. All the rest—15,800 soldiers—fell victims to privation and disease.—*London Medical Times & Gazette.*

